# The effect of audio-support on strategy, time, and performance on reading comprehension in secondary school students with dyslexia

**DOI:** 10.1007/s11881-021-00246-w

**Published:** 2021-11-19

**Authors:** C. A. N. Knoop-van Campen, D. ter Doest, L. Verhoeven, E. Segers

**Affiliations:** 1 Behavioural Science Institute, Radboud Universiteit, Nijmegen, Netherlands; 2 Radboud Universiteit, Nijmegen, Netherlands; 3 Instructietechnologie, Universiteit Twente, Enschede, Netherlands

**Keywords:** Audio-support, Dyslexia, Eye tracking, Reading comprehension strategies, Reading comprehension

## Abstract

The use of adequate reading comprehension strategies is important to read efficiently. Students with dyslexia not only read slower and less accurately, they also use fewer reading comprehension strategies. To compensate for their decoding problems, they often receive audio-support (narration written text). However, audio-support linearly guides readers from beginning to end through texts, possibly hindering the use of reading comprehension strategies in expository texts and negatively impacting reading time and reading comprehension performance. We examined to what extent audio-support affects reading comprehension strategies, reading times, and reading comprehension performance in 21 secondary school students with dyslexia and 22 typically developing controls. Participants were provided with three types of assignments (summarizing, open-ended questions, statement questions) in each condition (written text with and without audio-support). SMI RED-500 eye tracker captured eye movements during reading. The standard deviation of the weighted fixation duration times on the three paragraphs was considered indicative of the disparity of readers’ attention within the text. Following a discrimination based on experts’ reading behavior and hand-coded validation, these scores visualized whether students used the intensive reading strategy (reading whole text) or selective reading strategy (focusing on part of the text). In open-ended assignments, students divided their attention more over the whole text instead of focusing on one specific part when audio was added. In addition, audio-support increased reading time in students with and without dyslexia in most tasks, while in neither of the tasks audio-support affected reading comprehension performance. Audio-support impacts reading comprehension strategy and reading time in all students.

## Introduction

Students with dyslexia often receive audio-support via narration of the written text to compensate for their lack of accurate and fluent decoding. However, students with dyslexia not only read slower and less accurately; they also use fewer reading comprehension strategies (Chevalier et al., 2017). Audio-support guides readers linearly — from beginning to end — through texts, which possibly hinders the use of more dynamic reading comprehension strategies for expository texts like scanning headers, searching for keywords, etc. This may also impact reading time and comprehension negatively. To understand how audio-support during reading comprehension assignments affects reading comprehension strategies, reading times, and reading comprehension performance, we compared the effects of reading comprehension tasks with and without audio-support in secondary school students with dyslexia as compared to their typically developing peers.

### Reading comprehension strategies

Reading comprehension is a crucial skill for students’ school success and plays an essential role in further academic development (Murnane et al., 2012). According to the Simple View of Reading (Hoover & Gough, 1990), reading comprehension is the product of language comprehension and decoding. As children over time become more fluent decoders, they can devote more attention to comprehension. Reading comprehension comprises the understanding of words in sentence context and the understanding of these sentences in order to obtain new information. A mental construction of the meaning of the text is then built, and new information is acquired from the passages being read (Perfetti & Stafura, 2014). As such, reading comprehension can be defined as the ability to construct meaning from interaction with text (Snow, 2002).

Different reading comprehension strategies can be used to construct meaning from a written text. Such strategies are not the same as reading skill. Although the distinction between reading skill and reading strategy in the literature can be called inconclusive (Afflerbach et al., 2008; Liu, 2010), there is consensus on three issues: (i) strategies are reader-oriented; skills are text-oriented; (ii) strategies represent conscious decisions; skills are deployed unconsciously; and (iii) strategies, unlike skills, represent a response to a problem (Liu, 2010, p. 154, as cited in Urquhart & Weir, 1998). Strategies can thus be seen as deliberate actions that a reader executes in order to achieve an established reading objective (Afflerbach et al., 2008; Manoli & Papadopoulou, 2012).

When intentional control is needed, reading behaviors are seen as reading comprehension strategies and not as text reading-only (Afflerbach et al., 2008; Manoli & Papadopoulou, 2012). In education, students are often provided with expository texts with goal-directed reading assignments as response to a problem (e.g., making a summary or answering a text-based question). Reading comprehension strategies to address such assignments can be divided into two overarching types: selective reading and intensive reading (Afflerbach et al., 2008; Liu, 2010). The choice of one of the two reading comprehension strategies depends to a large extent on the reading objective (Guthrie & Mosenthal, 1987; Urquhart & Weir, 1998). The *selective reading strategy* can be defined as “The performance of learners who have the goal of detecting a specific subset of information within a relatively wide array of information that is displayed for visual inspection” (Guthrie & Mosenthal, 1987, p. 283) and is used to quickly understand the core and essentials of a text. A selective reading strategy is used when the reader is looking for specific information (e.g., the answer to a question) without having to understand the rest of the text (Guthrie & Mosenthal, 1987; Krishnan, 2011): finding pertinent information within a wider array of information. All parts of the text that do not contain the information required are skipped (Liu, 2010). The *intensive reading strategy* can be defined as “The different operations where the reader attempts to extract complete meanings within or beyond sentences right up to the level of the entire text” (Katalayi & Sivasubramaniam, 2013, p. 877). This strategy is used when making a summary of the text or for reading to recall a text (Krishnan, 2011; Liu, 2010). When applying the intensive reading strategy, the reader makes the decision to read the entire text and use all information in the text (Liu, 2010; Urquhart & Weir, 1998

An important aspect of successful reading comprehension is the extent to which students can regulate their use of reading comprehension strategies (Andreassen et al., 2017). Students who use more different reading comprehension strategies generally achieve higher reading comprehension scores (Salmerón et al., 2017). Via the measurement of eye movements during reading, reading strategies can be identified in an objective way (Van Gog & Jarodzka, 2013). The eye movements (fixation and saccades) during reading can be condensed into a scan path, providing an overview of where, how long, and in what order a student looked at the text. The selective reading strategy would be characterized by most fixations on one section of the text in particular, while an intensive strategy would show eye movements across the whole text (following Liu, 2010). Previous research into concept-mapping showed difference on reading in their scan paths after learning strategy instruction (Liu, 2014). Duggan and Payne (2011) studied scanning behavior on the Internet for information and showed that more fixations on relevant information led to higher reading comprehension scores. Eye tracking may thus help to better understand reading processes and how they are related to comprehension. However, existing research has not yet reached the stage of providing an easy and transparent measure for defining the reading comprehension strategy used, which could improve the accessibility of an objective measure of reading comprehension strategies.

### Reading comprehension in students with dyslexia

The chosen reading strategy depends on the predefined reading objective and is determined by the reading skills of the individual reader. A group of students with impaired reading skills are students with dyslexia. Dyslexia is considered to be a phonological-based reading disorder in which the relation between orthography and phonology is hampered, not caused by a lack of intelligence or education (Lyon et al., 2003). Decoding remains effortful, and reading speed remains slow. In line with the Simple view of Reading (Hoover & Gough, 1990), these decoding problems may have a severe impact on students’ reading comprehension (Lyon et al., 2003; Roitsch & Watson, 2019). As ineffective decoding is considered to have a negative effect on the available resources for understanding the text (Smythe, 2005; Wolf & Katzir-Cohen, 2001), reading comprehension can be compromised, because the student can only process a certain amount of information at once. Students with dyslexia also tend to use fewer reading comprehension strategies than their peers without dyslexia (Chevalier et al., 2017). For example, Kirby and colleagues (Kirby et al., 2008) showed that students with dyslexia experienced more difficulty in deriving key points from a text than students without dyslexia, which would point towards problems with using the selective reading strategy. Even though research has shown that the reading behavior (decoding) on an eye movement level is different in students with dyslexia compared to controls (e.g., De Luca et al., 2002; Hutzler & Wimmer, 2004), there is hardly any research in this population on reading comprehension strategies when reading a longer text.

Even though there are only few studies on reading comprehension strategies in students with dyslexia, related research indicates that poor readers use less reading comprehension strategies (Haberlandt et al., 1989; Hawelka et al., 2015) and have problems choosing the most efficient reading strategy to the corresponding reading objective (e.g., Anastasiou & Griva, 2009; Karimi & Shabani, 2013; Lau & Chan, 2003; Singhal, 2001).

Role of audio-support in digital reading compensation.

For students with dyslexia in higher education, reading software is the most commonly provide tool to compensate for their reading problems (Ghesquière et al., 2011). This audio-support consists of reading software (Ghesquière et al., 2011) and/or text-to-speech software (Draffan et al., 2007) both facilitating the computer to read (parts of) the learning material out load. Many audio-support tools can be activated on-demand at the word, sentence, paragraph, or whole text level. However, the most common default setting is to read the text from the beginning to the end (unless the student interrupts the system).

Wood et al. (2018) conducted a meta-analysis on the effects of the use of text-to-speech (digital read-aloud in addition to written text) on general reading comprehension. Results showed that audio-support has the potential to improve comprehension in students with decoding difficulties. Recent research in primary school children with dyslexia found no additional benefit of the use of audio-support on comprehension (Knoop-van Campen et al., 2018, 2019). Furthermore, in university students (with and without dyslexia), it was demonstrated that audio-support resulted in different reading behaviour (more focus on the supporting illustration and less on the written text) and, moreover, lead to lower comprehension (Knoop-van Campen et al., 2020).

### Present study

The extent to which audio-support impacts secondary school students remains unclear. Audio-support may increase reading time as narration (speaking the words out loud) may cost more time than silent reading (Knoop-van Campen et al., 2020). As audio-support linearly — from start to finish — guides readers through a text, this could impede the use of reading comprehension strategies such as scanning headlines and searching for keywords. When reading to recall a complete text, intensive reading (reading the whole text) is an effective strategy to learn (Krishnan, 2011; Liu, 2010) and aligns with reading a text alongside the voice-over in audio-support. However, selective reading could often be a more suitable strategy to answer a question about a part of the text. Audio-support which leads a reader linear through a text may then hamper effective strategy use. Using eye tracking, differences between reading with and without audio-support can be made visible. It remains unclear to what extent audio-support affects reading time and performances. Therefore, the research question in the present study was: What is the impact of audio-support on reading comprehension strategies, reading time, and reading comprehension performance in secondary school students with dyslexia as compared to typically developing peers?

Students were provided with three types of task-based reading assignments (summarizing assignments/open-ended assignments/statement assignments) with and without audio-support. The summarizing task was meant to elicit the intensive strategy; the open-ended assignment and statement assignment were meant to elicit the selective reading strategy (Liu, 2010). It was expected that audio-support would have no effect on the use of the intensive reading strategy (as elicited in summary assignments), but would have a negative effect on the use of selective reading strategies (as elicited in open-ended and statement assignments). As typically developing students were expected to use more selective reading strategies, the impact of audio-support would be especially visible in this group compared to students with dyslexia. In a similar vein, audio-support was expected to have a negative effect on reading time (mainly expected in open-ended and statements assignments) and would be especially visible in typically developing students as they have better (faster) reading skills than students with dyslexia.

As a result, audio-support was also expected to have a negative effect on reading comprehension performance and would be especially visible in open-ended and statements assignments and in typically developing students.

## Method

### Participants

A total of 43 eighth grade students (21 students with dyslexia; 22 typically developing peers) from six schools across the Netherlands participated in this study. The schools were all situated in residential areas with an average SES. Informed active consent was obtained from parents, students, and schools. This study was approved by the Ethics Committee of the Faculty of Social Sciences of our university.

All students with dyslexia were officially diagnosed with dyslexia, mostly during primary school (76% below the age of 12 years old), by a certified child psychologist following the clinical Protocol Dyslexia Diagnosis and Treatment (Blomert, 2005), which is a guide to diagnosing, indicating, and treating clients with dyslexia. To be eligible for this clinical assessment, one has to score either in the lowest 10% of reading or in the lowest 15% of reading *and* the lowest 15% on spelling, for three test measurements in a row despite extra efforts from school to improve these scores.

Only monolingual children were included. The control group was selected from the same classrooms as the students with dyslexia. Of the initial 43 participants, seven participants had to be excluded due to a tracking ratio below 70% (*N* = 2 dyslexia, *N* = 5 typically developing). Tracking ratio is the percentage of time that eye movements were measured during the exposition of the stimuli. One participant could not be included due to missing data as a result of computer malfunction (*N* = 1 dyslexia). Tracking ratio of the included group was 95.86% (*SD* = 4.75%).

The remaining 35 students (*M*_age_ = 13.20, *SD* = 0.54; 16 female) were included in the data analyses: 18 students with dyslexia (*M*_age_ = 13.17, *SD* = 0.51; 9 female) and 17 typically developing peers (*M*_age_ = 13.24, *SD* = 0.75; 7 female). The students with and without dyslexia did not differ in age, *t*(33) = 0.32, *p* = 0.167, *d* = 0.11. Three students had a(n additional) diagnosis (dyslexia, *N* = 1; anxiety disorder, *N* = 1 ADD; typically developing, *N* = 1 PDD-NOS). In line with their diagnosis, students with dyslexia scored lower on word reading fluency and pseudo word reading fluency (*M* = 64.33, *SD* = 11.89, and *M* = 63.00, *SD* = 14.31, respectively) than the control group (*M* = 85.59, *SD* = 12.18, and *M* = 87.12, *SD* = 15.39, respectively): word reading, *t*(33) = 5.23, *p* < 0.001, *d* = 1.77, pseudo word reading, *t*(33) = 4.81, *p* < 0.001, *d* = 1.62. The groups did not differ on their grade for Dutch language at the end of seventh grade, *t*(32) = 1.23, *p* = 0.098, *d* = 0.42. Students with dyslexia achieved an average of 6.4 (*SD*: 0.65) out of 10, the controls achieved an average of 6.8 (*SD*: 0.92).

### Design

To elicit reading comprehension strategies, participants were provided with three types of task-based reading assignments (summarizing assignments/open-ended assignments/statement assignments) in two conditions (written text with and without audio-support). The summarizing task was meant to elicit the intensive strategy; the open-ended assignment and statement assignment were meant to elicit the selective reading strategy (Liu, 2010). By measuring eye movements during the tasks, students’ reading comprehension strategies were deduced (see the “3” section). In order to interpret the strategies of the secondary school students as selective or intensive, a group of adult expert-readers also made the assignments. Following a discrimination based on experts’ reading behavior and hand-coded validation, these scores visualized whether students used the intensive reading strategy (reading whole text) or selective reading strategy (focusing on part of the text).

In the present study, a within-subject design in which all students received all task types both with and without audio-support was used. As such, all participants made the same six reading comprehension assignments, but varied in the order in which the assignments were presented and which assignments had audio-support. This was determined by a randomized-block-design: the experiment consisted of two blocks with three reading assignments each (before the start of each block a practice assignment was provided). One block had audio-support, the other one did not. Each block contained a summary assignment (text 1 or 2), an open-ended assignment (text 3 or 4), and a statement assignment (text 5 or 6), in which texts 1, 3, and 5 were always offered together in the same block and text 2, 4, and 6 in the other block. The order of type of assignments within blocks (summary/open-ended/statement), block (1/2), and audio-support (yes/no) were randomized leading to 24 possible randomizations.

### Materials

#### Task-based reading assignments

Students received six task-based reading assignments consisting of three types of assignments (summarizing assignment; open-ended assignment; statement assignment) in each condition (written text with and without audio-support). The different assignments elicited intensive reading strategies (filling in a summary: information from the whole text is needed) or selective strategies (open-ended and statement questions: answer is situated in one paragraph). The expository text in the assignments had general topics appropriate to the level of eighth grade students with on average 349 words per text (*SD* = 64) (see Table 1).

**Table 1:** Overview of the task-based reading assignments

	Title and subheadings	Assignment type	Location of the answer	Elicited reading strategy
Text 1	T: Power of bamboo S1: The knowledge of the past S2: Today's knowledge	Summary	Whole text	Intensive
Text 2	T: 100% slave free chocolate S1: Slave trade in the chocolate chain S2: Towards 100% slave-free chocolate	Summary	Whole text	Intensive
Text 3	T: Trucks without driver S1: A positive future S2: Less enthusiastic	Open-ended	2nd paragraph	Selective
Text 4	T: What can you do against climate change? S1: Causes of climate change S2: Behavioural change	Open-ended	3th paragraph	Selective
Text 5	T: Could it be a bit less? S1: CO2 emissions S2: Regulations	Statement	2nd paragraph	Selective
Text 6	T: Delft Hyperloop S1: No air resistance and obstacles S2: And the winner is…	Statement	3th paragraph	Selective

*T* = title, *S1* = subheading one, *S2* = subheading two

In order to ensure the reliability of the assignments, a *pilot study* was performed before conducting the present research. Fifty-two secondary school students (*M*_age_ = 12.79, *SD* = 0.64; 26 female) were asked to complete 17 task-based reading assignments. These assignments were based on Kachergis and colleagues (Kachergis et al., 2018) and were slightly revised by a Dutch specialist (also Dutch teacher) to accurately match the abilities of the students in the present study. Following the pilot, the most suitable assignments were selected for the present study: all chosen assignments had mean scores between 0.3 and 0.7 (indicating good discriminatory power, see Field (2013)) and were selected in pairs per type (in such a way that comparable assignments were available in the condition with and without audio-support). In total, six assignments were selected (with mean scores of *M*_p-value_ = 0.49, *SD*_p-value_ = 0.14): two summarizing, two open-ended assignments, and two statement assignments.

The assignments were presented to participants by means of slides on a computer screen (1680 × 1050 pixels with a font size of 20 pixels). Each assignment started with the reading comprehension question on the first slide, then on the next slide the text was presented, and on the third slide, the question was repeated and students could fill in their answer. Students were not allowed to move back to previous slides.

In the audio-condition, the written text was also read by a professional voice-over (female voice). The voice-over started to read the text when the text-slide appeared. Students were able to control the audio (e.g., pausing, repeating, skipping parts, and selecting were in the text the audio should read further), but hardly used this option. More than 72% of the students did not control the audio at all. Of the students who did control the audio, this was limited to on average less than one time per text (*SD* = 0.80). Before the start of each condition, students were presented a practice assignment, in order for them to understand how the assignments were build (first–second-third slide) and to practice with operating the audio-support.

For the *summarizing* assignments, students had to fill in several missing words into a short summary of the main text. On slide one, students would read (in Dutch) “After reading the text, fill in the missing 13 words at the right place in the summary. The summary and words are provided after the written text.” On the third slide, students saw the short summary with blank spaces and right next to it the words they had to fill in in the blank spaces, with the instruction “In the summary below, fill in the 13 missing words.” Students were given one point for each word correctly filled in in the summary. The total number of points that could be obtained was fourteen for text 1 and thirteen for text 2. For both texts, the scores were converted into a 0–1-point scale.

Concerning the *open-ended* assignments, the students were asked to provide examples based on information gathered from the text. On slide one, students would read (in Dutch) “Which advantages of self-steering trucks are mentioned in the text? (open question).” On the third slide, exactly the same was stated after which students could type in their answer. Students could obtain 1 point for each argument that was correctly mentioned (4 in text 3 and 5 in text 4). The answers were scored by two different researchers. The reliability between the assessors was excellent (inter-class correlation question 3: 1.00; inter-class correlation question 4: 1.00). For both texts, the scores were converted to a 0–1-point scale.

Regarding the *statement* assignments, students had to indicate whether a statement was true or false. On slide one, students would read (in Dutch) “Indicate whether the statement is true or false: The team of students at TU Delft had designed the fastest vehicle.” On the third slide, exactly the same was stated, and students could tick “true” or “false.” When the participants provided the correct answer, they were awarded one point. If nothing was entered, the participants received zero points.

The summary assignments encouraged students to read the entire text, as the required information to answer these questions was spread out throughout the whole text. This type of assignments corresponds to the intensive reading strategy. For the assignments in which the required information was located in one specific paragraph (open-ended and statement assignments), the selective reading strategy is most appropriate as this strategy optimizes the readers efficiency in finding the right information. An overview of the types of assignments is presented in Table 1. Additionally, the location of the answer and the most appropriate reading strategy are displayed.

**Fig. 1:**
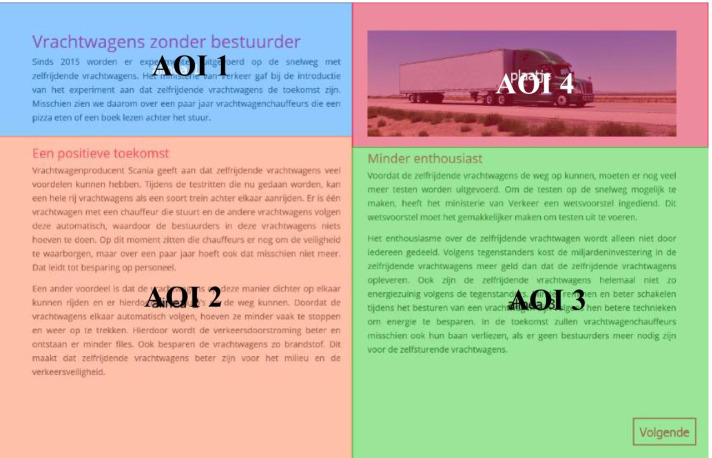
AOI layout

Each text included three paragraphs and a decorative image: paragraph 1 was at the top left of the screen; paragraph 2 at the bottom left, the image at the top right; and paragraph 3 at the bottom right. In the first paragraph, the subject of the text was briefly introduced, while in the next two paragraphs, the subject was explained in more detail. The texts consisted of an average of 348 words (*SD* = 48, range: 285–465). The texts were divided into four areas of interest (AOIs): AOI 1 (paragraph 1), AOI 2 (paragraph 2), AOI 3 (paragraph 3), and an image, AOI 4 (see Fig. 1, four boxes were added to indicated the four AOI’s). The text AOIs contained 116 words on average (*SD* = 35, range: 53–180).

#### Reading measures

To check for group differences between the students with and without dyslexia, (pseudo) word reading was examined. This was measured by means of the Een-Minuut-Test (EMT) [one-minute test] (Brus & Voeten, 1999) and the Klepel (Van den Bos et al., 1994). In both tests, students had to read out loud as many (pseudo) words as possible from a list of words on a card within 1 and 2 min, respectively. The score is the number of words a participant read correctly.

### Apparatus

The SMI RED500 was used to monitor and record eye movements of participants during reading. The eye tracker was controlled with the SMI IView program, and for the data analyses, SMI Experiment Center BeGaze 3.7 was used. Students were seated in front of the eye tracker at approximately 65 cm (*SD* = 3.89) (eyes — eye tracker). A nine-point calibration was used for the calibration. Calibration continued until a value < 1.00 was reached or when the lowest possible value was achieved based on the calibration difficulties of that specific participant. On average, students needed three times calibrating (*M* = 2.77, *SD* = 1.72). Calibration values varied with means of 0.67 (*SD* = 0.41) and 0.77 (*SD* = 0.36).

### Reading comprehension strategies

Students’ reading comprehension strategies were based on fixation duration times extracted from BeGaze (standard settings: saccade detection minimum duration of 22 ms, peak velocity threshold of 40°/s, minimal fixation duration of 50 ms, data channel right eye).

First, *weighted fixation duration times* were calculated to provide an indication of the amount of time a student spend on each paragraph, taking into account the differences in word length of the paragraphs. These weighted fixation durations were calculated by, first, determining the fixation duration per word for each AOI per text. Then, the fixation duration per word per AOI was divided by the total fixation duration of the three text-AOI’s and multiplied by 100 (to provide percentages). The higher this percentage in the event of a specific AOI was, the more attention has been paid to this paragraph (ranging from 0% when the paragraph was not looked at to 100% when the student only looked at that paragraph).

Then, to provide an indication of the distribution of a readers’ attention within the text, so whether a reader divided his/her attention over the whole text or focused on a specific part of the text, the standard deviation of the weighted fixation duration times was calculated (*disparity score*) and used for analyses. This disparity score could vary between 0 (when the attention was exactly evenly divided over the three AOI’s: as the standard deviation of 33%, 33%, and 33% is 0) and 57 (when the attention was 100% focused on one AOI: as the standard deviation of 0%, 0%, and 100% is 57).

**Fig. 2:**
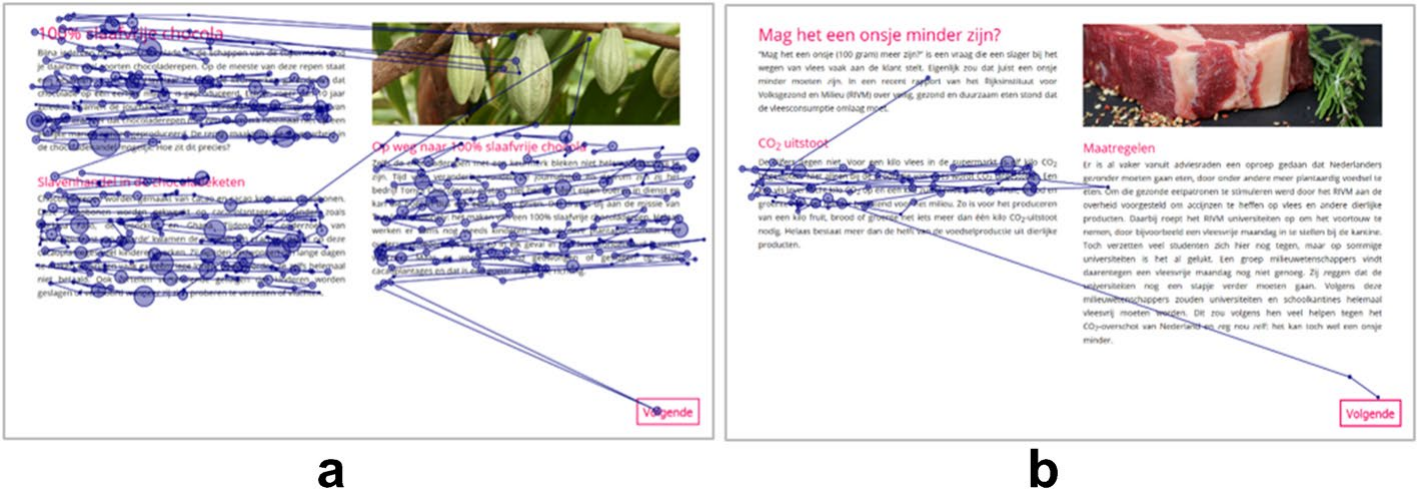
**a** Example of an intensive reading strategy. **b** Example of a selective reading strategy

As adults represent the end state of reading capacity, a group of 10 adult expert-readers^1^ made all six assignments without audio-support to establish baseline strategies used in the various assignments. Their disparity scores were calculated. Following a discrimination based on the experts’ reading behavior, these scores visualized the intensive and selective reading strategy (see Fig. 2a and b) in order to facilitate interpretation. In the four assignments which elicited the selective reading strategy, experts had a mean disparity score of 26.65 (*SD* = 17.24). In the two summarizing assignments, experts had a mean disparity score of 8.20 (*SD* = 3.51) with a maximum of 13.88. Following this maximum, the border line for the intensive reading strategy was set at a disparity score of 14 and below — attention could be equally distributed over the paragraphs with a standard deviation of zero (33% per paragraph), but could also be, for example, 48%, 31%, and 21% as in the case of the limit value of 14. Gaze displays with a disparity score above 14 were coded as selective reading strategies — attention was focused on a specific part of the text.

To validate this discrimination, the gaze displays of the secondary school students were hand coded by two coders (second author and a Dutch teacher). The two coders had a 93% agreeability on the strategies (*κ*_mean_ = 0.809, *p* < 0.001), and the hand coding was comparable to the coding based on the border line of 14 (*κ*_mean_ = 0.746, *p* < 0.001).

### Procedure

Schools were recruited by e-mail, and when interested, the eligible students with dyslexia (and an equal amount of typically developing peers from the same class and gender) were invited to participate.

Testing was done by the first two authors and six undergraduates (supervised, and after training) in an individual setting in school. Data collection was performed according to a test protocol that described into detail: the procedure, instructions, eye tracker set-up, calibration, and the tasks. In total, the experiment lasted approximately 55 min. Data collection was done in the middle of the 8th grade.

### Data analysis

To examine the impact of audio-support on reading comprehension strategies, reading time, and reading comprehension performance in students with and without dyslexia, GLM repeated measures were conducted with condition (without audio/with audio) and assignment type (summary/open-ended/statement) as within-subjects-factors, and group (dyslexia/controls) as between-subjects-factor. This was done for reading comprehension strategies (disparity score), reading time (time in minutes), and reading comprehension performance (reading comprehension scores) with a fixed significance threshold of *p* < 0.05.

Even though distributions of some variables were slightly skewed (see Table 2), transformation did not improve the distributions. When the assumption of sphericity was not met (according to Mauchly’s test of sphericity), Greenhouse–Geisser or Huynh–Feldt was reported (see Field, 2013).

**Table 2:** Skewness and Kurtosis for all variables

	Reading comprehension strategies		Reading time			Reading performance	
	Skewness	Kurtosis	Skewness	Kurtosis	Skewness		Kurtosis
Summary assignment							
Text condition	1.40	.92	.01	− .12	− 1.42		1.43
Text-audio condition	1.95	3.71	− 1.79	2.93	− 1.52		1.49
Open-ended assignment							
Text condition	.61	− .50	.35	− .07	.04		− .81
Text-audio condition	1.40	1.10	− 1.02	.30	− 1.11		1.20
Statement assignment							
Text condition	.53	− .31	.23	− .81	− 3.09		8.03
Text-audio condition	.98	.18	− .65	− 1.18	− 2.53		4.69

## Results

The means and standard deviations of students’ reading comprehension strategies, reading times, and reading comprehension performance scores for both groups with and without audio are presented in Table 3.

**Table 3:** Reading comprehension strategies, time, and performance per assignment type, group, and condition

	Reading comprehension strategies					Reading time					Reading performance				
Assignment type/group		Text condition		Text-audio condition			Text condition		Text-audio condition			Text condition		Text-audio condition	
	*N*	*M*	*SD*	*M*	*SD*	*N*	*M*	*SD*	*M*	*SD*	*N*	*M*	*SD*	*M*	*SD*
Summary assignment															
Dyslexia	18	9.76	6.30	7.82	6.73	18	2.38	.64	2.54	.63	16	.90	.14	.94	.13
Controls	17	14.30	11.55	17.14	16.54	17	1.70	.87	2.19	.70	16	.90	.14	.90	.14
Total	35	11.97	9.37	12.35	13.18	35	2.05	.82	2.37	.68	32	.90	.14	.92	.14
Open-ended assignment															
Dyslexia	18	22.71	16.03	11.32	12.55	18	2.23	1.12	3.14	.67	16	.56	.27	.55	.22
Controls	17	22.46	15.79	18.28	16.36	17	1.67	.52	2.53	1.29	16	.56	.27	.73	.19
Total	35	22.59	15.68	14.70	14.73	35	1.96	.92	2.84	1.05	32	.55	.27	64	.22
Statement assignment															
Dyslexia	18	18.08	11.76	14.55	12.46	18	1.72	.69	2.23	.60	16	.88	.34	.88	.34
Controls	17	22.37	14.31	20.61	15.98	17	1.25	.60	1.72	.87	16	.94	.25	.94	.25
Total	35	20.16	13.04	17.50	14.40	35	1.49	.68	1.98	.77	32	.90	.30	.91	.30

Reading comprehension strategies: disparity score, range 0–57. Reading time: minutes. Reading comprehension performance: scores range 0–1

### Reading comprehension strategies

Regarding reading comprehension strategies, results showed main effects of condition, *F*(1, 33) = 4.83, *p* = 0.035, *η*^*2*^_*p*_ = 0.128, and of assignment type, *F*(1.71, 56.40) = 7.93, *p* = 0.002, *η*^*2*^_*p*_ = 0.194, but not of group, *F*(1, 33) = 2.72, *p* = 0.109, *η*^*2*^_*p*_ = 0.076. In addition, there was a significant interaction effect between condition and assignment type, *F*(1, 33) = 5.60, *p* = 0.024, *η*^*2*^_*p*_ = 0.148. Follow-up analyses per assignment type showed that for open-ended assignments students had a lower disparity score in the text-audio condition than in the text condition, *F*(1, 33) = 8.61, *p* = 0.006, *η*^*2*^_*p*_ = 0.207. For summarizing and statement questions, no statistical difference between the conditions were found (*F*(1, 33) = 0.10, *p* = 0.760, *η*^*2*^_*p*_ = 0.003, and *F*(1, 33) = 0.79, *p* = 0.381, *η*^*2*^_*p*_ = 0.023, respectively).

**Fig. 3:**
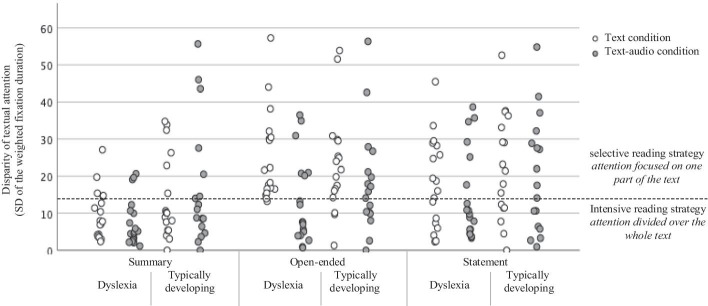
Disparity (distribution) of students’ attention over the written text per assignment type, group, and condition

The disparity scores indicating the distribution of students’ attention over the written text, are visualized in Fig. 3. A horizontal dotted line is drawn, based on the expert reading behaviour, dividing the disparity scores into intensive reading strategy (below 14) and selective reading strategy (above 14).

### Reading time

Regarding reading time, results again showed main effects of condition, *F*(1, 33) = 26.47, *p* < 0.001, *η*^*2*^_*p*_ = 0.445 and assignment type, *F*(1, 33) = 23.81, *p* < 0.001, *η*^*2*^_*p*_ = 0.419, and now also a main effect of group, *F*(1, 33) = 10.21, *p* = 0.003, *η*^*2*^_*p*_ = 0.236. There were no significant interaction effects. Students had higher reading times (examined the text longer) in the text-audio condition compared to in the text condition. Students had higher reading times in summarizing and open-ended assignments compared to the statement assignments. Students with dyslexia had higher reading times than typically developing students.

### Reading comprehension performance

Regarding *reading comprehension performance*, results showed a main effect of assignment type, *F*(1, 30) = 77.29, *p* < 0.001, *η*^*2*^_*p*_ = 0.720, but not of condition, *F*(1, 30) = 0.89, *p* = 0.353, *η*^*2*^_*p*_ = 0.029, or group, *F*(1, 30) = 1.77, *p* = 0.193, *η*^*2*^_*p*_ = 0.056. There were no interaction effects. Students had higher reading comprehension performance scores in the summarizing and statement assignments compared to the open-ended assignments.

## Discussion

We examined the impact of audio-support on (1) reading comprehension strategies, (2) reading time, and (3) reading comprehension performance in secondary school students with dyslexia as compared to their typically developing peers.

### Reading comprehension strategies

As expected, students showed mainly intensive reading in the summarizing assignments and mainly selective reading in the assignments in which the correct answer was located in a specific part of the text (open-ended and statement assignments) (e.g., Krishnan, 2011; Liu, 2010). In addition, we showed that audio-support indeed had a negative effect on the use of the selective reading strategy, which was visible in the open-ended assignments. In such type of assignments, students divided their attention more over the whole text (intensive reading strategy) instead of focusing on one specific part (selective reading strategy) when audio was added. This is in line with a Danish study in six students with dyslexia showing that the reading process changed when literacy technology (which included audio-support) was used (Svendsen, 2016).

The fact that with audio-support students in the open-ended assignments were more inclined to use the intensive reading strategy rather than the selective reading strategy indicates that the audio elicited less efficient reading behaviour (Krishnan, 2011; Liu, 2010). The question is whether students consciously applied the intensive strategy or that they were simply “dragged through the text” by the audio. After all, when a reader with audio-support applies a selective strategy, he/she interrupts the narration. Such a choice is a conscious one, while being guided by the audio can be an automatic reaction, especially when students have reading problems themselves. The audio-support could be controlled (switched on/off, moved, etc.) but this too required a conscious action on the part of the student; students hardly used this option. A possible reason why we only found a difference in strategy in the open-ended assignments and not in the statement questions, even though they both elicited the selective reading strategy, could lie in the fact that answering open-ended questions may require more mental effort than statement questions do (Gwizdka, 2008). Whereas the former requires to find and reproduce a coherent answer, the latter only requires to find and recall true/false.

In contrast to our expectations, we did not evidence differences between students with and without dyslexia in reading comprehension strategies or the impact of audio-support thereof. In contrast to other research on reading comprehension strategies at the word and sentence level between good and poor readers (e.g., Karimi & Shabani, 2013), this research shows that when examining overarching reading comprehension strategies, students with dyslexia show the same strategic reading behavior. Participants in the present study had a relative low time pressure. If there would be (considerable) time pressure, then a (faster) selective strategy could have paid off more (Krishnan, 2011).

### Reading time

In line with our expectations, audio-support affected the time students spent on the written text on most tasks: it increased reading time in students with and without dyslexia in open-ended and statement assignments. Even though students with dyslexia had higher reading times, as expected, see, e.g., Roitsch and Watson (2019), audio-support affected both groups equally. This is in line with Knoop-van Campen and colleagues (2020) who also showed that audio-support in learning tasks increased study time in university students with and without dyslexia, but contrary to similar research in primary school children who became faster with audio-support (Knoop-van Campen, et al., 2019). The impact of audio-support on reading times may change over time and possibly relates to reading proficiency. As audio-support was found to be especially beneficial for students with low reading skills (Dunsworth & Atkinson, 2007), for (relatively) good readers, the audio may be too slow. This could lead to a misalignment between the pacing of the audio and the student’s own reading pace.

Apparently, even the students with dyslexia could read faster than the audio. Although reading did cost them more time compared to their typically developing peers, audio also slowed them down. The tipping point where audio-support is no longer beneficial for efficient learning, thus seems to be when reading becomes faster than speaking; on average between the age of 11 (see Knoop-van Campen et al., 2018, 2019) and 13 years.

### Reading comprehension performance

In contrast to our expectations, audio-support did not affect students’ performance in any of the tasks. The Cognitive Load Theory (Paas et al., 2003) indicates that simultaneously presenting identical written and spoken text imposes an additional burden on the working memory (increased cognitive load) and can, therefore, hinder the absorption of information leading to lower reading comprehension performance. To what extent this applies to students with dyslexia is unclear, as audio-support may also alleviate cognitive load for these students as it lessens the burden on working memory due to effortless decoding (Gerbier et al., 2018).

Contrary to studies which showed that children with dyslexia have difficulty with reading comprehension (e.g., Casalis et al., 2013), the performance scores did not indicate differences in performance on the tasks. However, previous studies often involved primary school children. The students in the present study are older and more experienced in reading. They may have been able to compensate better for possible secondary reading comprehension problems (Bråten, et al., 2010).

The lack of impact of audio-support on task performance could partially be due to the difficulty level of some of the tasks. For example, in the summary assignments, the students were given a partially completed summary, in which they had to fill in words from a list. This is easier than writing a summary themselves, as more details need to be remembered when writing a summary (Smith, 1967). Moreover, in more challenging assignments, background knowledge would play a greater role in linking the word identification and word comprehension system (Perfetti & Stafura, 2014), costing more cognitive effort (Lau & Chan, 2003).

We did not evidence differences in reading comprehension performance between students with and without dyslexia or differences in the impact of audio-support on performance. In contrast to previous research (Smythe, 2005; Wolf & Katzir-Cohen, 2001), their poorer decoding skills did not lead to poorer comprehension of the text.

### Limitations and future research

Some limitations can be put forward. Firstly, group sizes were relatively small. However, by matching as much as possible on school and gender, and by using a within-subject design, we aimed to decrease environmental differences and increased reliability.

Second, despite good mean-values in the pilot study (within the desired range of 0.4 and 0.6, see Field, 2013), students in the experimental study scored high on the summarizing and statement assignments leading to ceiling effects. Future research could investigate whether performance is affected in more challenging tasks. When the texts and/or assignments become more difficult, students will have to use reading comprehension strategies (Afflerbach et al., 2008).

Third, merely based on the eye tracking data, it is not possible to distinguish whether an intensive strategy was indeed a conscious choice to read the whole text or whether it indicated only reading skills (see Afflerbach et al., 2008). However, as students had to answer the reading comprehension question (and all students did), their reading was goal-directed. We, therefore, considered their reading behaviour as strategic. Nevertheless, future research could incorporate (retrospective) think-aloud interviews (Paans et al., 2020) based on students’ eye-moves through the text. This could provide information on the level of consciousness of possible reading choices.

Fourth, we have examined students’ reading behaviour only on a global level, distinguishing between two general reading comprehension strategies. In future research, our classification system to differentiate between the intensive and selective reading strategy could be refined by including other aspects of reading behaviour.

Finally, as there is a large variation in disparity scores and thus the degree of attention within a text (see Fig. 3), it would be interesting to further examine, in a larger scaled study, the extent to which individual differences impact the relation between audio-support and strategy use. These measures could include, for example, general knowledge of reading comprehension strategies, compensation variables like motivation — as a high intrinsic motivation could compensate for reading problems (Polychroni et al., 2006; Singer, 2007), vocabulary (Keenan & Meenan, 2014), or background knowledge (O’Reilly et al., 2019).

### Practical implications and conclusion

In students with dyslexia, audio-support may increase engagement and involvement in the material (Grusky et al., 2020; Rahman et al., 2010; Sidhu & Mazura, 2011), increase confidence (Caute et al., 2018), and support reading stamina and motivation (Larson, 2015).

However, the present research also shows that there are some drawbacks to audio-support, especially in terms of time and reading strategy for open-ended questions. This does not mean that audio-support should be discouraged, as it can still compensate for decoding problems. Nevertheless, it would seem sensible to raise students’ awareness of, and actively support them in, the impact audio-support may have on their reading behavior (Svendsen, 2016). When students become aware that audio makes them read less efficiently, they may (learn to) use the audio more actively and, by doing so, increase their efficiency.

Regarding reading comprehension strategies in general, results show that many students do not use the most efficient strategy, even without audio-support. Afflerbach and colleagues (Afflerbach et al., 2008) point towards metacognitive instruction about how, why and when to use strategies as effective in education. Explicit instruction, modeling, and scaffolding appropriate reading comprehension strategies could support students in developing their reading comprehension strategies (Okkinga, et al., 2018).

To conclude, audio-support affected reading comprehension strategies in open-ended assignments towards more intensive reading strategies, in which selective reading would be considered to be more appropriate. In addition, audio-support increased the time students with and without dyslexia spent on their assignments across different tasks types, even though performance scores were similar. Audio-support thus impacts reading strategy and reading time in all students.

## Data Availability

Test protocol and stimuli are available on request.

## Footnotes

1 All expert-readers were raised monolingual and were not diagnosed with dyslexia. One expert could not be included because of a tracking ratio below 70%. The remaining nine experts were included in the data analysis (7 women; average age: 23.11 years, *SD*: 1.83). They had an average tracking ratio of 95.24% (*SD*: 4.68) and all scored excellently on the tests for fluent reading of words and pseudo words (*M*: 103.44, *SD*: 15.91; *M*: 103.89, *SD*: 9.03, respectively).
